# Industrial side streams as sustainable substrates for microbial production of poly(3-hydroxybutyrate) (PHB)

**DOI:** 10.1007/s11274-022-03416-z

**Published:** 2022-10-19

**Authors:** Elodie Vlaeminck, Evelien Uitterhaegen, Koen Quataert, Tom Delmulle, Karel De Winter, Wim K. Soetaert

**Affiliations:** 1 Bio Base Europe Pilot Plant (BBEPP), Rodenhuizekaai 1, Ghent, Belgium; 2 Centre for Industrial Biotechnology and Biocatalysis (InBio.be), Ghent University, Ghent, Belgium

**Keywords:** Biopolymer, Fermentation, Industrial side streams, Poly(3-hydroxybutyrate) (PHB), Second-generation feedstocks

## Abstract

Poly(3-hydroxybutyrate) (PHB) is a microbially produced biopolymer that is emerging as a propitious alternative to petroleum-based plastics owing to its biodegradable and biocompatible properties. However, to date, the relatively high costs related to the PHB production process are hampering its widespread commercialization. Since feedstock costs add up to half of the total production costs, ample research has been focusing on the use of inexpensive industrial side streams as carbon sources. While various industrial side streams such as second-generation carbohydrates, lignocellulose, lipids, and glycerol have been extensively investigated in liquid fermentation processes, also gaseous sources, including carbon dioxide, carbon monoxide, and methane, are gaining attention as substrates for gas fermentation. In addition, recent studies have investigated two-stage processes to convert waste gases into PHB via organic acids or alcohols. In this review, a variety of different industrial side streams are discussed as more sustainable and economical carbon sources for microbial PHB production. In particular, a comprehensive overview of recent developments and remaining challenges in fermentation strategies using these feedstocks is provided, considering technical, environmental, and economic aspects to shed light on their industrial feasibility. As such, this review aims to contribute to the global shift towards a zero-waste bio-economy and more sustainable materials.

## Introduction

While plastics have become almost indispensable with widespread applications in various sectors, their ever-increasing usage has severe environmental impacts. The carbon footprint of traditional fossil-based plastics amounts to 1.7 Gton CO_2_ eq. per year, or 4% of the global greenhouse gas emissions (Zheng and Suh 2019). In addition, after a service life of often less than one year, also their disposal has a disastrous impact on the environment as a staggering amount of 20 Mton of plastic ends up in the oceans every year (Borrelle et al. 2020). Although regulations are being put in place to reduce plastic usage, such as bans on single-use plastics, various industries are in need of alternative, more sustainable materials (Nielsen et al. 2020). In this context, polyhydroxyalkanoates (PHAs), which are polyesters that can be produced by prokaryotes from renewable resources, are witnessing a major surge of interest (Li et al. 2016). These biopolymers are known for their biodegradation under various conditions, including in marine environments (Kabir et al. 2020). Poly(3-hydroxybutyrate) (PHB) is by far the most occurring and extensively studied representative of the PHA family with material properties similar to conventional polypropylene (PP), hence, suitable for a wide range of applications (Kumar et al. 2020). In particular, its barrier properties result in a great potential for food packaging, while its biocompatibility makes PHB suitable for medical and pharmaceutical products (Koller 2014; Israni and Shivakumar 2019).

Nevertheless, despite its bio-based nature, interesting material properties, and excellent biodegradability, today’s annual PHB production is estimated at only 4 kton, whereas the global bioplastics market amounts to 2.4 Mton (Koller and Mukherjee 2022). Due to its relatively high production cost of approximately 3 €/kg, PHB currently struggles to be cost-competitive with established fossil counterparts such as PP with a market price of about 1 €/kg. In this respect, the choice of feedstock is crucial as it typically constitutes up to 50% of the total PHB production cost (Koller 2019). Although current commercial processes achieve high PHB production performances, they use high-purity first-generation substrates which are not only economically unfavorable but raise ethical concerns as well. Consequently, the use of industrial side streams as inexpensive and more sustainable feedstocks could offer a solution. This, however, entails new challenges related to the accessibility of the carbon source, the presence of impurities, and the restricted assimilation by PHB-producing microorganisms.

Therefore, this mini-review provides a comprehensive and critical overview of the use of industrial side streams for fermentative PHB production. First, the state-of-the-art PHB production processes using conventional, pure carbon sources are described as a benchmark. Afterward, a concise overview is provided of the industrial side steams, where their feasibility as an alternative feedstock is discussed based on the attained PHB production performance and required pre-treatments, taking into account technical, environmental, and economic considerations. Notably, this mini-review does not aim to cover the large number of carbon sources described in literature. In contrast, it rather puts focus on the most promising ones for PHB production, which were selected based on state-of-the-art research results considering the full spectrum of biomass side streams as well as C1 gas emissions. As such, it presents the most important inexpensive heterotrophic and autotrophic feedstocks to make PHB biosynthesis on a larger scale economically efficient, and, at the same time, upgrade waste streams and save resources of food and fodder relevance typically used in PHB production to date.

## State-of-the-art PHB production from first-generation biomass

The first PHB bioplastic was marketed over 30 years ago and the biopolymer has gained increasing attention ever since, leading to several main players on the PHB market to date, including Biomer (Germany), PHB Industrial S.A. (Brazil), Bio-On (Italy), and Tianan Biologic materials (China) (da Cruz Pradella et al. 2012). Their PHB production processes encompass an aerobic fermentation using high-performing PHB-producing bacteria, such as *Cupriavidus necator* (formerly known as *Ralstonia eutropha*), *Azohydromonas lata* (formerly known as *Alcaligenes latus*), and *Paraburkholderia sacchari*, which accumulate PHB intracellularly under conditions of physiological stress. The latter can be induced by the depletion of a nutrient essential for growth, such as nitrogen, while carbon is abundantly present (Koller 2018). Hence, to assure high PHB production, the fermentation process typically consists of two phases: (1) a growth phase to attain a high number of cells, and (2) a production phase to induce high PHB accumulation within the cells.

The carbon sources commonly used in today’s commercial PHB processes are mostly carbohydrates derived from first-generation biomass (Koller and Mukherjee 2022). As such, the fed-batch cultivation of *A. lata* on sucrose and *C. necator* on glucose, resulted in 99 and 139 g/L PHB at a rate of 5.0 and 3.1 g/(L∙h) PHB, respectively (Wang and Lee 1997a; Shang et al. 2003). Notably, wild-type *C. necator* H16 cannot assimilate glucose, hence, adapted strains should be used, such as *C. necator* NCIMB11599 which was used by Wang and Lee (1997a). Furthermore, also the highest reported PHB titers, amounting up to 161 g/L PHB, have been obtained from glucose by using recombinant *Escherichia coli* strains (Wang and Lee 1997b; Kahar et al. 2005). However, besides the fact that these purified carbohydrates are expensive and thus contribute to the high production cost, they are also derived from edible crops whose use is controversial in non-food applications and further present an environmental burden because of their intensive monoculture cultivation (Lips 2021). Recent advances focusing on the use of industrial side streams as alternative feedstocks could enable more economical as well as sustainable overall PHB production. Table 1 presents an overview of the most promising side streams and their use as a feedstock for PHB production, as well as the benchmark processes on first-generation carbohydrates.

**Table 1:** Overview of the most representative bioreactor studies for each category of feedstocks considering the treatment and performance of the PHB fermentation process.

Feedstock	Treatment	Substrate (S)	Strain	Biomass (g/L)		PHB (%)		Productivity (g/(L∙h) PHB)		Yield (g/g PHB/S)		Remarks		References		
**First-generation biomass - benchmark**																
		Sucrose	*A. lata* DSM1123	112		88		5.0		-				(Wang and Lee 1997a)		
		Glucose	*C. necator* NCIMB11599	208		67		3.1		0.38				(Shang et al. 2003)		
		Glucose	*E. coli* XL1-Blue ^a^	201		80		2.8		0.38				(Kahar et al. 2005)		
**Carbohydrate-containing industrial biomass side streams**																
Sugar beet molasses	Sucrose hydrolysis	Glucose, fructose	*C. necator* ATCC25207	29		53		0.3		0.13		Glucose accumulation hampered production		(Ertan et al. 2021)		
Bovine whey	Spray-drying, protein removal	Lactose	*E. coli* CGSC4401 ^a^	120		80		2.6		0.34				(Ahn et al. 2000)		
Waste potato starch	Liquefaction, saccharification	Glucose, maltose	*C. necator* NCIMB11599	179		53		1.5		0.22		Only glucose was converted		(Haas et al. 2008)		
**Lignocellulose-containing industrial biomass side streams**																
Wheat straw	Lignocellulose disruption, hydrolysis	Glucose, xylose, arabinose	*P. sacchari* DSM17165	146		72		1.7		0.22		Full carbohydrate conversion		(Cesário et al. 2014)		
**Lipid- and glycerol-containing industrial biomass side streams**																
Waste soybean cooking oil	None	Glycerides	*C. necator* H16	145		79		1.7		0.76				(Loan et al. 2022)		
Crude glycerol	Fatty acids removal, demethanolization	Glycerol	*C. necator* DSM545	105		63		1.4		0.30				(Mozumder et al. 2014)		
**Direct and indirect use of industrial C1 gas emissions** ^**b**^																
CO_2_		CO_2_, H_2_, O_2_	*C. necator* DSM545	44		61		0.17		-		Growth phase on glycerol, O_2_ limitation hampered production		(Garcia-Gonzalez et al. 2015)		
Methane		CH_4_, O_2_	*Mehylocystis sp.* GB14	60		51		1.3		-		High O_2_ supply and extensive cooling required		(Wendlandt et al. 2010)		
C1 gas-derived intermediates		Acetic acid	*C. necator* DSM545	79		74		0.93		0.23				(Vlaeminck et al. 2022)		
		Methanol	*Pseudomonas sp.* K	206		66		0.78		0.18				(Suzuki et al. 1986)		
		Ethanol	*E. coli* W3110 ^a^	77		47		0.31		0.27				(Sun et al. 2020)		

^a^ Recombinant strain further modified for PHB production.

^b^ As industrial C1 gases have not been used for PHB synthesis to date, the cited results in this part include the utilization of synthetic gas mixtures and pure liquid intermediates. Therefore, no treatment is indicated.

## PHB production from industrial biomass side streams

### Carbohydrate-containing side streams

Since today’s commercial PHB production is largely based on carbohydrates, carbohydrate-containing side streams appear as straightforward alternative substrates. As such, molasses is the main side stream of the sugar industry and contains up to 40% of sucrose (Teclu et al. 2009). However, most PHB-producing organisms, including the model organism *C. necator*, do not have hydrolase enzymes to metabolize sucrose, thereby requiring a hydrolysis treatment prior to fermentation (Dalsasso et al. 2019). Depending on the applied hydrolysis method, which can be chemical (acid or alkaline) or enzymatic, various impurities can be formed that may negatively affect the PHB biosynthesis. Typically, enzymatic methods result in the formation of fewer inhibitors and are more environmentally friendly, yet the relatively high cost of enzyme utilization should also be considered (Sen et al. 2019). Nevertheless, similar results have been obtained on enzymatic and acid hydrolyzed molasses in fed-batch fermentations using *C. necator*, amounting to 12 and 15 g/L PHB, respectively (Table 1) (Dalsasso et al. 2019; Ertan et al. 2021). Alternatively, to reduce treatment costs, recombinant strains have been constructed enabling direct conversion of sucrose into PHB. Recently, such *C. necator* strain has successfully been cultivated in a batch fermentation on sugarcane molasses, resulting in complete extracellular sucrose hydrolysis followed by utilization of all resulting glucose and fructose and producing 20 g/L biomass containing 83% PHB (Jo et al. 2021). However, in all three cited cases, one carbohydrate was consumed preferentially, eventually resulting in the accumulation of glucose or fructose in the fed-batch fermentations, which inhibited higher PHB production. This was avoided by Kiselev et al. (2022) where only the batch phase was performed on molasses hydrolysate, while pure glucose was used for feeding during the fed-batch phase. Although this led to the production of 85 g/L biomass containing 80% PHA at 1.2 g/(L∙h) PHA, such a strategy thus requires the addition of an expensive first-generation feedstock. Additionally, trace amounts of propionic acid and valeric acid in the molasses led to the synthesis of poly(3-hydroxybutyrate-*co*-3-hydroxyvalerate) (PHBV) containing 0.5 mol% 3HV, instead of the PHB homopolymer (Kiselev et al. 2022). Although PHBV is a copolymer with interesting characteristics, consistent product purity and quality cannot be assured in this case because the 3HV content is unpredictable as it varies according to the composition of the feedstock.

Next to molasses, also whey is a carbohydrate-containing industrial side stream that has been described as a suitable substrate for PHB production and is generated in large quantities during cheese-making processes (Amaro et al. 2019). In contrast to the highly concentrated molasses, whey contains only around 5% lactose, along with a high amount of proteins and fats (Asunis et al. 2020). Therefore, before its use as a microbial feedstock, concentration and purification are required. Additionally, whey is often spray-dried to obtain an even higher lactose concentration and increase stability, which translates into significant treatment costs (Koller et al. 2007). Although the lactose disaccharide can be metabolized by some wild-type PHB-producing organisms, including *Methylobacterium sp.* and *A. lata*, this has yielded relatively low amounts of PHB (Nath et al. 2008; Berwig et al. 2016). Alternatively, *C. necator* strains have been modified to utilize lactose, while lactose-utilizing *E. coli* strains have been modified to produce PHB (Lee et al. 1997; Povolo et al. 2010). The cultivation of such *E. coli* resulted in the production of 120 g/L biomass containing 80% PHB at 2.6 g/(L∙h) PHB (Table 1) (Ahn et al. 2000). Although this competes with state-of-the-art processes, highly concentrated spray-dried whey was used, hence, still resulting in a high overall PHB production cost. To avoid extensive whey pre-treatment, also whole whey has been studied as a potential substrate, yet this brings important technical drawbacks, including sterility issues. In this regard, the use of halophilic bacteria growing under high salinity has recently been proposed as a promising way to prevent contamination. However, metabolic tools to allow lactose conversion by these strains are currently lacking, while other challenges arise, including treatment of the saline effluent and corrosion of fermentation equipment (Obruča et al. 2022).

Finally, also starch can be considered as an interesting carbohydrate-containing side stream, as it is present in residues from root and tuber crops generated by food and feed processing industries. Starch contains amylose and amylopectin carbohydrate polymers, which both consist of glucose units. Therefore, after rehydration of the starch (liquefaction), the polymers first need to be hydrolyzed (saccharification) to obtain a carbohydrate mixture. This mixture of mainly glucose and maltose can be used as a substrate for PHB production (Salimi et al. 2019). Haas et al. (2008) reported the use of waste potato starch hydrolysate and obtained 95 g/L PHB at 1.5 g/(L∙h) PHB, however, maltose accumulated as this could not be assimilated by the *C. necator* strain, thereby causing a decreased conversion and significant carbon loss (Table 1).

### Lignocellulose-containing side streams

Lignocellulosic biomass is the most ubiquitous renewable carbon source in the world. This is reflected in many agricultural residues, such as straw or stover from rice, barley, wheat, and corn, as well as forestry residues, such as woodchips, branches, and sawdust. Lignocellulosic side streams are also abundant in various other industries, including brewer’s spent grain, paper waste, and de-oiled press cakes. Therefore, lignocellulose appears as a very promising substrate for PHA production, as reviewed by Obruča et al. (2015). The main challenge in the use of lignocellulosic biomass is the need for extensive pre-treatment to break its recalcitrant, complex structure of cellulose, hemicellulose, and lignin. Afterward, hydrolysis of the (hemi)cellulose is required to yield readily fermentable carbohydrates. Apart from the significant impact on the total cost, the choice of pre-treatment and hydrolysis strategy (physical, chemical, or enzymatic) dictate the final composition of the hydrolysate in terms of carbohydrates, organic acids, lignin derivatives, and potential other microbial inhibitors, hence, also influence the subsequent PHB production process (Vigneswari et al. 2021).

Interestingly, it was observed that through directed adaption and evolution, *C. necator* can become resistant to and even metabolize some of these contaminants including organic acids, furfurals, and aromatic compounds from acid-treated sugarcane bagasse hydrolysates, thereby producing 11 g/L biomass containing 57% biopolymer (Yu and Stahl 2008). However, because of the presence of propionic acid and valeric acid, PHBV was formed instead of PHB, which can be undesirable as explained above. Another challenge when using lignocellulosic biomass includes the utilization of all different carbohydrates, thereby striving for complete carbon conversion. In this context, *P. sacchari* strains capable of metabolizing glucose, xylose, and arabinose simultaneously, even in the presence of inhibitors, have gained attention (Lopes et al. 2011). As such, and by the implementation of a controlled feeding strategy to overcome carbon catabolite repression, 146 g/L biomass containing 72% PHB could be produced from wheat hydrolysates with a productivity of 1.7 g/(L∙h) PHB, thereby reaching the highest PHB production performance to date on lignocellulosic biomass (Table 1) (Cesário et al. 2014). Besides, efforts have also been focusing on increasing the carbon conversion when using *C. necator*, by modifying it to metabolize xylose, though so far only at a low performance (Kim et al. 2016). Moreover, next to (hemi)cellulose hydrolysates, also aromatic lignin derivates have been investigated as potentialcarbon sources for microbial PHB production (Tomizawa et al. 2014). Since today less than 2% of the available lignin side streams are converted, this could have a high potential, yet it is a complex side stream consisting of many types of aromatic compounds. Because the conversion of lignin to PHB remains low, with the highest reported results amounting to only 2.1 g/L PHB, further strain engineering advances will be required to evaluate if lignin could indeed be a suitable sustainable carbon source for PHB production (Li et al. 2019; Ganesh Saratale et al. 2021).

In conclusion, as opposed to carbohydrate-rich feedstocks, lignocellulosic side streams require intensive pre-treatment steps prior to their hydrolysis, thereby increasing the total cost of the process. Interestingly, the number of process steps and costs can be reduced by simultaneous saccharification and fermentation (SSF) (Kawaguchi et al. 2016). This can be beneficial for the conversion of lignocellulose as well as carbohydrate polymers. For example, by feeding lignocellulosic cereal mash to a bioreactor containing glucoamylase as well as the PHB-producing strain *Halomonas boleviensis*, 26 g/L PHB was produced, however, the hydrolytic efficiency of the enzymes for this SSF was found to be lower than for separate hydrolysis and fermentation (García-Torreiro et al. 2016). In this respect, it should be taken into account that SSF is a challenging process demanding optimization of various parameters to enhance both the hydrolytic efficiency and the PHB production performance. Besides SSF, also consolidated bioprocessing (CBP) has recently been investigated for starch as an intensified, integrated approach. It involves the construction of strains producing enzymes to hydrolyze the starch polymers extracellularly, thereby avoiding the enzyme cost. As such, Brojanigo et al. (2022) used an amylolytic *C. necator* strain which directly produced 13 g/L biomass containing 43% PHB from broken rice waste, demonstrating the potential of CBP. Nonetheless, similar to SSF, further advances are required to improve the overall process performance.

### Lipid- and glycerol-containing side streams

Lipid-containing industrial side streams mainly constitute waste cooking or frying oils from the food industry as well as from household waste. These waste oils primarily consist of mono-, di-, triglycerides, and some fatty acids, which can all be used as feedstocks for PHB production. Promising results have been obtained on untreated waste soybean cooking oil, producing 145 g/L biomass with 79% PHB at a rate of 1.7 g/(L∙h) PHB (Table 1) (Loan et al. 2022). Interestingly, these results are similar to the results obtained with the same *C. necator* strain on pure soybean oil (Kahar et al. 2004). This technology is currently being established by the company Nafigate (CZ), using *C. necator* for the conversion of waste cooking oils into PHB (Nafigate Corporation 2021). In contrast, when using waste frying oil, lower performances have been obtained, amounting to only 1.5 g/L PHA (Vastano et al. 2019). This is most likely caused by the presence of inhibiting compounds, such as peroxides, terpenes, phenols, and furans, which are formed during frying as a result of the high temperatures catalyzing oxidation reactions (Talan et al. 2020). Furthermore, while the substrate conversion yield for most feedstocks ranges from 0.2 to 0.4 g/g PHB/S, up to 0.8 g/g PHB/S has been obtained when using oils (Table 1). This is due to their high carbon content and the direct incorporation of fatty acids into the polymer, which can thus also lead to the formation of PHA copolymers, for example, poly(3-hydroxybutyrate-*co*-3-hydroxyhexanoate) (PHBHHx) is often formed when using palm oil (Riedel et al. 2012; Sato et al. 2015).

To date, vegetable oils are widely used in biodiesel production for which they are transesterified with methanol, thereby generating an aqueous side stream containing 50% glycerol, unesterified fatty acids, and methanol. After separation of the fatty acids and demethanolization, crude glycerol remains as the major by-product and has extensively been investigated as feedstock for PHA production (Koller and Obruča 2022). Next to about 85% glycerol, crude glycerol also contains relatively high levels of contaminants, including methanol, salts, and fatty acids. However, since purification of such glycerol streams is almost ten times more expensive than pure glycerol, direct usage of crude glycerol is crucial (Chol et al. 2018). Moreover, it was found that particularly the presence of NaCl in crude glycerol inhibits PHB accumulation, which can be circumvented by modifying the biodiesel manufacturing process so that K_2_SO_4_ is present instead (Mothes et al. 2007). The best results on crude glycerol were achieved by Mozumder et al. (2014), amounting to 105 g/L biomass containing 63% PHB at 1.4 g/(L∙h) PHB, which is in line with the performance obtained on pure glycerol (Table 1).

## PHB production from industrial C1 gas emissions

### Direct utilization of C1 gases: CO_2_, CO, CH_4_

Next to biomass side streams, also industrial C1 gas emissions have been explored as alternative feedstocks for PHB production in the framework of the upcoming carbon capture and utilization (CCU) technologies. Indeed, some well-known PHB-producing organisms, including *C. necator* and *Methylobacterium extorquens*, contain the Calvin cycle, allowing them to fix carbon dioxide (CO_2_) by using hydrogen (H_2_) as electron donor and oxygen (O_2_) as final electron acceptor (Lee et al. 2021). However, the development of a gas fermentation process to obtain CO_2_-derived PHB is highly challenging, mainly because: (1) the transfer of the gaseous substrates to the culture is low, (2) the Calvin cycle is known to be energetically inefficient demanding large amounts of H_2_, and (3) the required gas mixture is potentially explosive when the minimal oxygen concentration (MOC) of 4.3 vol% is exceeded (Molnarne and Schroeder 2017). Hence, these limitations hamper the supply of sufficient O_2_ and H_2_, which can lead to low biomass production and decreased overall productivity. Nevertheless, Tanaka et al. (1995) reported the production of 91 g/L biomass containing 68% PHB at 1.5 g/(L∙h) PHB using CO_2_ as the sole carbon source. Remarkably, this involved an increase of the O_2_ supply by sparging 6.9 vol% O_2_ and a lab-scale bioreactor with an exceptional and industrially less relevant design to increase the gas transfer. Another strategy has been proposed by Garcia-Gonzalez et al. (2015) and involves a growth phase on crude glycerol, while CO_2_ was used only for PHB production with 2.8 vol% O_2_. This resulted in 46 g/L biomass with 61% PHB at only 0.17 g/(L∙h) PHB, as the low O_2_ availability nonetheless limited the PHB production phase (Table 1). Conclusively, this shows that increased oxygen supply (potentially above the MOC) is required to attain high PHB production from CO_2_, which encompasses some major technical challenges including the use of specialized equipment to increase the gas transfer and assure safe operating conditions (Lambauer and Kratzer 2022). Alternatively, instead of H_2_, light can be used as inexpensive energy-source by phototrophic cyanobacteria, however, additional research is required to overcome remaining hurdles regarding their cultivation in photobioreactors, as has been reviewed by Yashavanth et al. (2021).

Moreover, it should be noted that all aforementioned results were obtained using pure synthetic gases. When considering industrial gas emissions, the composition can vary, and also gas treatment should be taken into account to remove impurities that could inhibit growth (Dhakal and Acharya 2021). In fact, industrial syngas, which is emitted by among others the steel and cement industry, contains primarily carbon monoxide (CO), next to H_2_ and CO_2_. Hence, in order to convert syngas, *C. necator* has been modified to oxidize CO to CO_2_ which can be used in the Calvin cycle, thereby increasing carbon conversion and PHB production. However, also O_2_ addition is required which still limits the total process performance (Heinrich et al. 2018). Alternatively, though still in its infancy, anaerobic syngas-utilizing strains, which efficiently assimilate CO directly through the Wood-Ljungdahl pathway, have been modified to produce PHB (Flüchter et al. 2019). Notably, syngas can also be derived from biomass gasification, which is mainly performed on difficult-to-treat lignocellulosic biomass as this is considered a simpler way to tap into the available carbon and allow direct fermentation, as compared to intensive pre-treatment and purification techniques (Rodionova et al. 2022).

Finally, also methane (CH_4_), which is largely emitted by agriculture, mining, oil, and gas industries, could be used as a gaseous carbon source as it can be converted by naturally occurring PHB-producing methanotrophic bacteria. Interestingly, since CH_4_ is less explosive than H_2_, more O_2_ can be added thereby allowing higher biomass concentrations and productivities. As such, Wendlandt et al. (2010) reported the production of 60 g/L biomass containing 51% PHB at 1.3 g/(L∙h) PHB using a *Meythylocystis* strain (Table 1). However, due to its reduced nature, the microbial consumption of methane has a high O_2_ demand, and the resulting exothermic oxidation reaction requires extensive cooling, which both significantly contribute to the final cost of the process (Levett et al. 2016). Nonetheless, Newlight Technologies (US) and Mango Materials (US) have started to commercialize methane-based PHB production at a relatively small scale of 50 and 5 ton/y, respectively (Muthuraj et al. 2021; Koller and Mukherjee 2022).

### Indirect utilization of C1 gases through liquid intermediates

To overcome the bottlenecks related to the utilization of industrial C1 gas emissions through aerobic gas fermentation, recent advances have presented their indirect utilization via a two-stage strategy. In the first stage, the gaseous carbon is converted into a simple one- or two-carbon liquid intermediate, which is then used as the substrate for a second stage, the PHB production fermentation. For example, Al Rowaihi et al. (2018) reported the electrochemical reduction of CO_2_ into formic acid, which was subsequently assimilated by *C. necator* producing 13 mg/L PHB. Similarly, Hwang et al. (2020) produced formic acid by biological conversion of CO and used *M. extorquens* for the subsequent conversion producing 0.10 g/L PHB. Notably, when using these wild-type strains, a large fraction of the supplied formic acid needs to be metabolized to CO_2_ in order to supply sufficient energy for PHB production, resulting in an overall inefficient CCU process (Bar-Even et al. 2012). In this respect, the development of novel synthetic routes enabling direct formate incorporation could offer a solution, while also improving PHB production (Claassens et al. 2020). Alternatively, better results have been obtained using acetic acid, amounting to 79 g/L biomass containing 74% PHB at 0.93 g/(L∙h) PHB, thanks to its more efficient metabolization towards PHB (Table 1) (Vlaeminck et al. 2022). Since acetic acid can also efficiently be produced from CO_2_ and CO by acetogenic bacteria in an anaerobic gas fermentation, this gives rise to an interesting two-stage fermentation. This challenging process has been developed for other high-value products, whereas so far only a proof-of-concept has been delivered for PHB production (Hu et al. 2016; Molitor et al. 2019).

In addition, although the execution of such a two-stage process has not been demonstrated to date, also methanol and ethanol have been suggested as interesting intermediates (Claassens et al. 2019). Methanol can be produced efficiently from the chemical reduction of CO_2_ with H_2_ and converted into PHB by methylotrophs. Suzuki et al. (1986) already reported a performant process using a *Pseudomonas* strain to produce 206 g/L biomass containing 66% PHB at 0.78 g/(L∙h) PHB from methanol (Table 1). However, as methanol is a hazardous compound, additional measures and thus costs should also be taken into account. In contrast to methanol, ethanol has only recently been investigated as an alternative sustainable feedstock for PHB production. Sun et al. (2020) reported the construction of an *E. coli* strain to convert ethanol into PHB and reported the production of 77 g/L biomass containing 47% PHB at 0.31 g/(L∙h) PHB (Table 1). This could be an interesting route as, up to now, the acetogenic conversion of syngas into ethanol is the sole gas fermentation process that is performed commercially on an industrial scale, namely by Lanzatech (US) (Fackler et al. 2021).

Furthermore, next to the valorization of C1 gas emissions into PHB, this indirect utilization strategy could also be implemented for biomass side streams. Here, syngas or volatile fatty acids (VFAs) can be generated as intermediates by gasification or by anaerobic acetogenic digestion, respectively. The latter has recently been investigated as a valuable route for PHB production from difficult-to-treat lignocellulosic streams, as well as from wastewater from various industries, yet so far still at low performance and prone to unpredictable copolymer formation because of the variable mixture of VFAs (Al Battashi et al. 2021; Amadu et al. 2021).

## Conclusion and outlook

PHB is a promising bioplastic with a broad range of applications, however, to date, its production cost is limiting widespread utilization. As the carbon source contributes to approximately 50% of the total cost and the sustainability of currently used carbon sources is questionable, attention is drawn to low-cost and more environmentally friendly substrates, such as industrial side streams. Therefore, this mini-review presents a critical evaluation of the use of these side streams as substrates for sustainable PHB production and compares this to state-of-the-art processes using first-generation substrates, taking into account technical, economic, and environmental considerations.

As concerns the carbohydrate and lignocellulosic side streams, strategies that eliminate upstream hydrolysis steps, such as the use of recombinant strains, SSF, or CBP, appear beneficial to reduce the overall costs, yet further advances will be required to increase their PHB production performance. Especially lignocellulosic side streams have been presented as attractive feedstocks because of their abundance and low cost, though the extensive pre-treatment required to break their recalcitrant structure cannot be overlooked. In contrast, waste cooking oils, as well as crude glycerol, can be used with no or limited pre-treatments and have shown to be competitive substrates for PHB production, particularly the former owing to its high conversion yield. Looking at future prospects, however, it should be noted that the supply of crude glycerol is vulnerable as this heavily depends on the demand for biodiesel.

Besides industrial biomass side streams, also industrial C1 gas emissions have been considered as alternative substrates for PHB production. They benefit from the attractive environmental advantage of being a CCU technology, which also entails an economically interesting perspective given the increasing emission taxes. Concerning CO_2_ fixation, additional H_2_ needs to be provided, thereby substantially increasing the feedstock cost. Nonetheless, considerable progress is being made to lower the cost of renewable electricity and establish large-scale green H_2_ production. Although the utilization of real industrial gas emissions has not yet been investigated, promising results have been obtained using synthetic gas streams. Here, the safe and sufficient supply of O_2_ was identified as the main bottleneck. This could be circumvented by an innovative best-of-both-worlds strategy involving the indirect utilization of C1 gases through liquid intermediates, allowing for efficient C1 gas fixation as well as PHB production. Future research will be required to conclude if this challenging two-stage process could indeed be technically as well as economically feasible.

In conclusion, several highly-promising side streams were presented with the potential to establish a more sustainable PHB production process by combining: (1) optimized and minimal pre-treatment, (2) a suitable (engineered) PHB-producing strain, and (3) implementation of an advanced fermentation strategy. Nevertheless, further advances in these three aspects, as well as others, such as PHB purification and application, could still improve the overall process performance. Furthermore, whereas all cited results, including some competitive with the current state-of-the-art processes, have been obtained on a lab-scale, scale-up studies will be crucial to demonstrate the industrial performance of the complete process.
